# Comparison of Intraocular Pressure Measurements Using Three Different Methods (Goldmann Applanation Tonometry (GAT), Corvis ST, and iCare) Following Penetrating Keratoplasty

**DOI:** 10.3390/jcm13237046

**Published:** 2024-11-22

**Authors:** Tatiana Tziola, Argyrios Tzamalis, Spyridon Koronis, Panagiotis Garitsis, Ioannis Tsinopoulos, Nikolaos Ziakas

**Affiliations:** 1 2nd Department of Ophthalmology, School of Medicine, Faculty of Health Sciences, Aristotle University of Thessaloniki, 54124 Thessaloniki, Greece; tziolatatiana@gmail.com (T.T.); spyridonkoronis@gmail.com (S.K.); pan.garitsis@gmail.com (P.G.); itsinop@auth.gr (I.T.); nikolasziakas@gmail.com (N.Z.); 2 Postgraduate Master Program “Ocular Surgery”, School of Medicine, Aristotle University of Thessaloniki, University Campus, 54124 Thessaloniki, Greece

**Keywords:** penetrating keratoplasty, intraocular pressure, Goldmann applanation tonometer, iCare tonometer, Corvis ST, biomechanical IOP, central corneal thickness, sutures, antiglaucoma medication

## Abstract

**Background/Objectives:** Intraocular pressure (IOP) readings using three different methods (Goldmann applanation tonometry (GAT), Corvis ST, and iCare) were compared in patients who underwent penetrating keratoplasty (PK). **Methods**: An observational cross-sectional study with prospective recruitment of patients was conducted. IOP measurements were acquired using GAT, iCare, and Corvis (including both uncorrected IOP (CVS-IOP) and biomechanical IOP (bIOP)), and the agreement among methods was analyzed using Bland–Altman plots. Secondary outcomes included the influence of CCT, the number of sutures, the size of the corneal donor button, and the use of antiglaucoma topical medications on the IOP readings using the three methods. **Results**: Twenty-five eyes from 25 patients were included. The Bland–Altman analysis showed the narrowest limits of agreement (LoA) between GAT and bIOP (7.5 mmHg). The difference between iCare and GAT IOP showed a bias of 1.26 ± 3.8 mmHg, with increased variability in cases with more remaining sutures (*p* = 0.0079). A higher CCT was moderately associated with lower bIOP readings (*p* = 0.0067), but no significant impact of CCT on the difference in the IOP measurements between GAT and other tonometers was found. Additionally, there were no significant differences in tonometer readings based on the use of antiglaucoma medications or the corneal donor button size. **Conclusions**: Good agreement was found between iCare, CVS-IOP, bIOP, and GAT-IOP readings with the comparison between GAT-IOP and bIOP resulting in the narrowest 95% LoA. The difference between the GAT-IOP and iCare readings tended to be influenced by the number of sutures at the graft–host interface. Higher CCT values were associated with lower bIOP readings; however, the differences in tonometer readings compared to GAT-IOP were not found to be influenced by CCT.

## 1. Introduction

Elevated intraocular pressure (IOP) after penetrating keratoplasty (PK) is one of the major threats to vision due to optic nerve damage or endothelial cell decompensation and subsequent corneal graft failure [1,2,3]. A meta-analysis carried out in 2017 showed that approximately one in five eyes that undergo PK will present with ocular hypertension or glaucoma in the postoperative period [4]. Early postoperative trabecular meshwork dysfunction in eyes that have undergone PK can result from inflammation, retained viscoelastic, or angle distortion due to surgical techniques, while tight or uneven suturing, formation of peripheral anterior synechiae, and prolonged use of high-potency corticosteroids increase the risk of late-onset elevated IOP [1,5,6]. The relationship between lowering IOP and the progression of both functional and structural glaucomatous injuries is not always linear. Nevertheless, each millimeter of mercury (mmHg) decrease in IOP can be important for preserving visual function and preventing further damage to the optic nerve [7,8]. The need for reliable post-PK IOP measurements is, therefore, imperative.

The Goldmann applanation tonometer (GAT, Haag-Streit, Koeniz, Switzerland) is widely regarded as the gold standard for IOP measurement in clinical practice [9]. Even though it has shown variable reproducibility in measuring IOP in normal eyes [10,11] it has demonstrated decent reliability in post-PK eyes [12]. Increased corneal surface irregularity and altered corneal geometry in the early postoperative period, however, may pose technical challenges for obtaining accurate GAT IOP measurements [13]. Moreover, alterations in corneal biomechanical properties such as lower corneal hysteresis and corneal resistance factor, lower rigidity, higher deformation amplitude, graft–host interface mechanics, and marked corneal astigmatism, as well as increased central corneal thickness, can further impact the accuracy of these measurements [14,15,16,17,18,19,20]. Additionally, repeated GAT measurements, especially during the initial postoperative phase, can potentially harm the fragile transplanted corneal epithelium [14].

Alternative tonometry methods have been developed to overcome these obstacles. Rebound tonometry (RT), such as iCare (Tiolat Oy, Helsinki, Finland), is a non-contact method used to measure intraocular pressure (IOP). It is based on the principle of measuring the rebound velocity of a small probe that makes brief contact with the cornea. It is particularly useful in cases where corneal irregularities, such as those found in post-PK eyes, make direct contact tonometry difficult or less reliable [21]. Several studies have shown a good correlation between iCare readings and GAT in normal eyes, though, in most studies, they tend to be consistently slightly higher, with the deviation increasing further in eyes with thicker corneas [22,23,24]. RT showed good reproducibility and accuracy of the IOP measurements compared to manometric studies in ex vivo human eyes [22].

Corneal Visualization Scheimpflug Technology (Corvis ST, CST, Oculus, Wetzlar, Germany) has recently been developed as an innovative non-contact tonometry technique. It utilizes a high-speed Scheimpflug camera system to precisely measure intraocular pressure (IOP) by dynamically analyzing corneal deformation in response to an air puff [25]. Corvis ST, apart from the standard GAT-correlated IOP (CVS-IOP) provides information on a biomechanically compensated IOP (bIOP). The bIOP algorithm incorporates factors such as CCT and age in addition to deformation response parameters to adjust for the effect of stiffness on IOP measurements [25,26,27]. Compared to manometric measurements of the interior of ex vivo human eyes, bIOP provided estimates very close to the true IOP without correlating to the CCT [28].

Corvis ST has previously been used to assess in vivo the biomechanical properties of post-keratoplasty eyes [20,29]. The time and radius values at the highest concavity were found to be significantly reduced in post-PK eyes [29] compared to the normal population, suggesting an impaired corneal structural integrity [20,29]. In eyes with all sutures removed at the graft–host interface, the deformation amplitude was not significantly different from that of normal controls [29]. However, the deformation amplitude was greater in post-PK eyes when compared to normal eyes, suggesting lower corneal rigidity in these eyes. This was attributed to insufficient stromal wound healing at the graft–host interface or possibly to abnormalities in the peripheral host cornea [20].

The purpose of this study was to compare IOP readings using three different tonometry methods: applanation tonometry using GAT, rebound tonometry with iCare, and non-contact tonometry utilizing Corvis ST in patients that underwent PK in a tertiary hospital. Additionally, the influence of CCT, the number of sutures, and the use of anti-glaucoma drops on tonometry readings were assessed. To the best of our knowledge, this is the first study to compare IOP measurements obtained using the Corvis ST with those from GAT and iCare in post-PK eyes.

## 2. Materials and Methods

### 2.1. Study Population

This is a cross-sectional comparative study with prospective recruitment of patients. Patients having undergone penetrating keratoplasty, who were examined in the cornea clinics of the Ophthalmology Department, in Papageorgiou General Hospital, a tertiary hospital, and a Cornea Transplant Center, in northern Greece, between May 2023 and June 2024, were included in this study. Indications for penetrating keratoplasty were pseudophakic bullous keratopathy (*n* = 9), previous graft rejection or failure (*n* = 6), keratoconus (*n* = 4), corneal opacification from various causes (*n* = 3), Fuchs Endothelial Dystrophy (*n* = 2), and previous corneal trauma (*n* = 1). Thirteen of the eyes were pseudophakic, and one eye had previously undergone glaucoma surgery (trabeculectomy and an Ahmed valve). The PKs were performed by three different surgeons. In twenty-one cases, 16 interrupted sutures were used, in two cases, 17 interrupted sutures were used, and in the remaining three cases, 8 interrupted sutures and 1 continuous suture were used. Additional inclusion criteria were age over 18 years and the ability to provide informed consent for participation in the study. Exclusion criteria included signs of graft rejection or failure, any current central epithelial defects, or a history of prolonged inflammation. Corneal edema increases the corneal thickness and hydration, which reduces the corneal rigidity and decreases the resistance to applanation, leading to lower measured IOP values; therefore, these cases were excluded from the study [16,19]. The study adhered to the tenets of the Declaration of Helsinki. All data presented were collected as part of routine clinical practice without any changes to standard patient care. The prospective study protocol was approved by the Institutional Review Board and Ethics Committee of the Aristotle University of Thessaloniki, Papageorgiou General Hospital (Ethic Approval Code: 307/24 Ethic Approval Date: 11 May 2024).

The patients were examined during morning office hours in the cornea clinics of the outpatient department during their follow-up visits after PK. The examination included the Corrected Distance Visual Acuity (CDVA), IOP measurement using GAT, Corvis, and iCare with patients in a seated position, a slit lamp examination, and measurement of the CCT using Corvis ST. The same examiner performed the GAT IOP and iCare measurements, while a different clinician performed the Corvis ST measurements. Two different measurements were obtained with GAT: one in the steepest and one in the flattest meridian. This is a standard practice in our clinic to address the challenges in IOP measurements caused by marked astigmatism in post-PK eyes [30,31]. The analysis was conducted using the mean of these two measurements (GAT-IOP).

During the measurement process with the iCare tonometer, the probe was positioned vertically to the central corneal plane. A set of six continuous measurements was obtained by the device, which then automatically analyzed the data and displayed the mean IOP value used for this analysis. The standard deviation of the measurements was used to determine the precision of the readings. The iCare tonometer provides four types of error bars—null, low, medium, and high—based on the standard deviation. In our study, only IOP results with null error bars were recorded, indicating a higher level of reliability and precision. If measurements were associated with error bars other than null, another measurement was performed to ensure accurate and consistent IOP assessment. A minimum 10-minute break between the applications of GAT and iCare was observed to eliminate measurement bias from aqueous outflow [32].

Corvis ST IOP measurements were performed only once in each eye. This approach was based on the previous literature, which suggested good repeatability and reproducibility of IOP readings with this device [33,34]. Due to the device’s different location, away from the outpatient department, these readings were taken within the next half-hour. Both non-corrected IOP and bIOP, obtained using Corvis ST, were recorded, along with the CCT. All tonometers were calibrated per the manufacturer’s guidelines.

All patients underwent a routine ophthalmological examination, as part of their standard post-operative follow-up visit. A slit lamp examination of the anterior segment was performed, and the number of remaining sutures between the corneal graft and the host was documented. Fundus examination in mydriasis was carried out only when deemed necessary by the clinicians. The demographic data collected included age, sex, topical medications, as well as the date and indication for penetrating corneal transplantation.

### 2.2. Statistical Analysis

The primary outcome of the study was the IOP readings obtained using GAT, Corvis ST, and iCare as well as the correlation between these measurements and GAT-IOP. Secondary outcomes included the influence of CCT, the number of sutures, and the use of antiglaucoma topical medications on IOP readings with the three different methods. Data were analyzed using r studio (Posit Software, PBC), version 2023.03.0+386.

Bland–Altman plots were constructed to evaluate the agreement between the different methods, comparing the difference between IOP readings from the iCare and Corvis and the GAT-IOP. The differences between GAT-IOP and the IOP measurements obtained from all other tonometers were tested using a paired *t*-test. To assess the relationship between tonometry readings and CCT, we conducted a correlation analysis using Kendall’s Tau correlation coefficient. The same test was also applied to evaluate whether a relationship exists between the number of sutures or the donor corneal button size and the tonometry readings. The Kruskal–Wallis test was used to evaluate the influence of anti-glaucoma drops on IOP measurements. A *p*-value of <0.05 was considered statistically significant. Descriptive statistics are reported as the mean ± standard deviation (SD).

## 3. Results

Twenty-five eyes from 25 patients were enrolled in the study and included in the analysis. Demographic data for the patients are presented in Table 1. The patient ages ranged from 44 to 81 years, with a mean age of 66.1 ± 12.2 years. Fifteen of the patients were female. The mean CDVA at the time of examination was 0.74 ± 0.5 logMAR. On the examination date, nineteen of the eyes had 10 or more sutures (mean 14.6 ± 2.1), two had six sutures, and all three still had the running suture. The mean time between grafting and the day of our measurements ranged from 1 to 35 months, with a mean of 14.4 ± 13.5 months. At the time of examination, all eyes exhibited a clear graft. Two cases of tight sutures were noted, and one case presented with severe superficial punctate keratopathy and a paracentral epithelial defect. 

Table 2 shows the mean IOP readings obtained from all tonometers. When compared to the GAT, the mean IOP readings from the other tonometers were not found to be statistically significantly different (*p* = 0.1127 for iCare, *p* = 0.1802 for CVS-IOP, and *p* = 0.3673 for bIOP).

**Figure 1:**
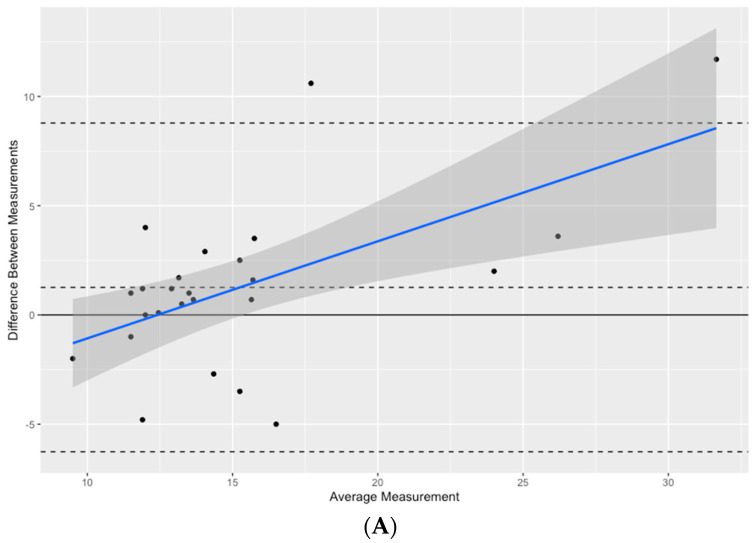

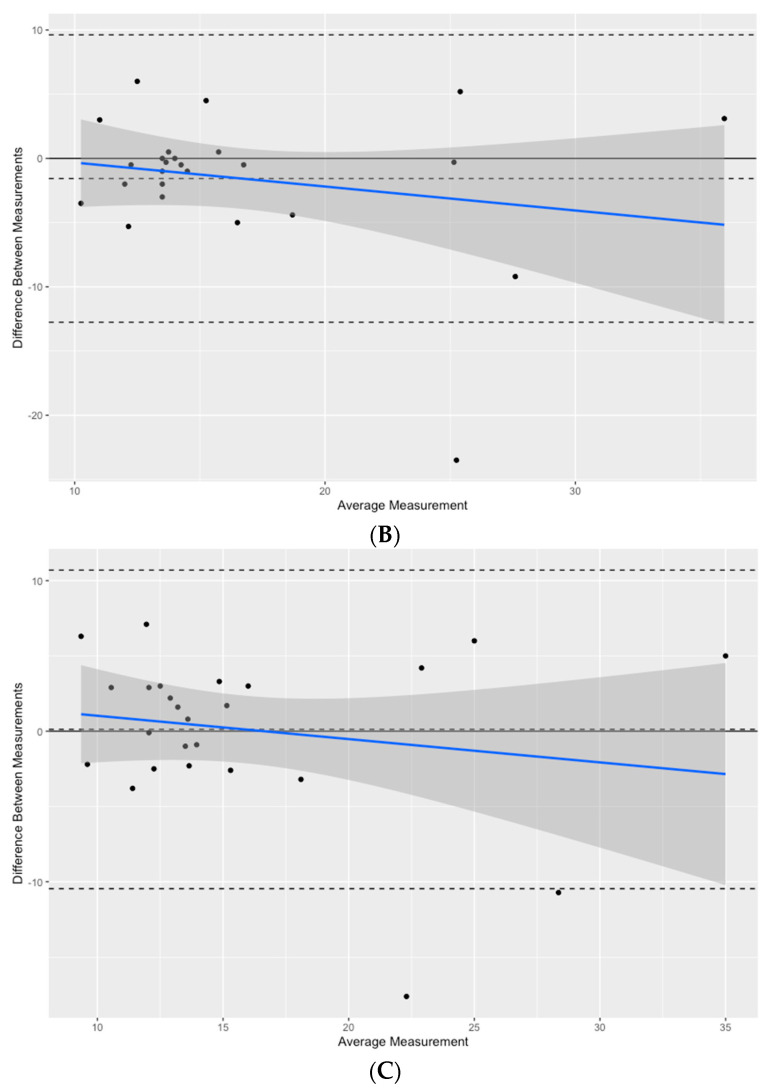
Bland–Altman plots illustrating the mean IOP difference (ΔIOP) between (**A**) iCare, (**B**) uncorrected IOP obtained by Corvis ST, and (**C**) biomechanically compensated IOP obtained by Corvis ST and Goldmann applanation tonometry. The distribution of ΔIOP is displayed on the *y*-axis, and the mean IOP values of the tonometers are displayed on the *x*-axis. A solid horizontal line represents the mean difference, whereas dashed horizontal lines depict the 95% limits of agreement and the zero-mean difference.

Figure 1A presents the Bland–Altman plot illustrating the agreement between GAT-IOP and iCare. On average, the iCare measurements were slightly higher than GAT-IOP, showing a bias of 1.26 ± 3.8 mmHg SD with a 95% confidence interval (CI) ranging from −0.32 to 2.84 mmHg, indicating potential variability. However, this difference was not statistically significant (*p* = 0.1138) The limits of agreement (LoA) ranged from −6.26 mmHg to 8.78 mmHg. The relatively wide spread of 15.05 mmHg between the upper and lower LoA indicates considerable variability between individual measurements. This suggests that while the methods generally agree, the differences can be substantial for certain measurements, particularly at extreme values. Finally, evidence of proportional bias is indicated by the slope of the regression line in Figure 1A: at lower IOP values (less than 14 mmHg), the iCare measurements were generally lower than GAT-IOP, while at higher IOP values, the iCare readings tended to be higher than GAT-IOP.

The mean CVS-IOP measurements were slightly lower than the GAT-IOP readings, as shown in Figure 1Β. IOP readings from the two instruments were also not found to be statistically different (*p* = 0.1824). The mean difference between CVS-IOP and GAT was −1.56 ± 5.7 mmHg with a 95% CI of −3.9 mmHg to 0.79 mmHg. The Bland–Altman plot of the difference between the two methods against their mean set the upper LoA at 9.6 mmHg and the lower LoA at −12.7 mmHg.

Finally, the mean difference between bIOP and GAT-IOP was 0.124 ± 5.3 mmHg, with a 95% confidence interval ranging from −2.1 to 2.3 mmHg, indicating minimal average disparity between the two methods (Figure 1C). The IOP readings from the two instruments also showed no statistically significant difference (*p* = 0.9095). The Bland–Altman plot yielded the narrowest 95% LoA among the methods compared, exhibiting a spread of 7.5 mmHg between the upper limit of agreement (14.6 mmHg) and the lower limit of agreement (6.8 mmHg).

The mean CCT in our cohort was 543.4 ± 62 μm. Kendall’s Tau rank correlation analysis revealed that the only statistically significant correlation between CCT and the various tonometer readings was with the bIOP (*p* = 0.0067). As the CCT increases, the bIOP shows a moderate negative correlation, with a Tau value of −0.391, indicating that higher CCT values are associated with lower bIOP readings. The findings are summarized in Table 3.

However, none of the correlations between the CCT and the differences in IOP measurements (ΔIOP) from the various tonometers compared to GAT-IOP were found to be statistically significant, as presented in Table 4.

Twenty-three eyes were on steroid drops, and five eyes were on anti-glaucoma medication at the time of examination. The Kruskal–Wallis tests indicated no significant differences in tonometer readings and the use of antiglaucoma medication (*p* = 0.5255 for GAT-IOP, *p* = 0.5224 for iCare, *p* = 0.4557 for CVS-IOP, and *p* = 0.4038 for bIOP).

The donor corneal buttons used ranged from 7.5 to 8.5 mm in diameter, with an average size of 7.89 ± 0.37 mm. No significant differences in tonometer readings were observed in relation to donor button size, as indicated by the Kruskal–Wallis tests. (*p* = 0.5835 for GAT-IOP, *p* = 0.5476 for iCare, *p* = 0.5532 for CVS-IOP, and *p* = 0.4963 for bIOP).

**Figure 2:**
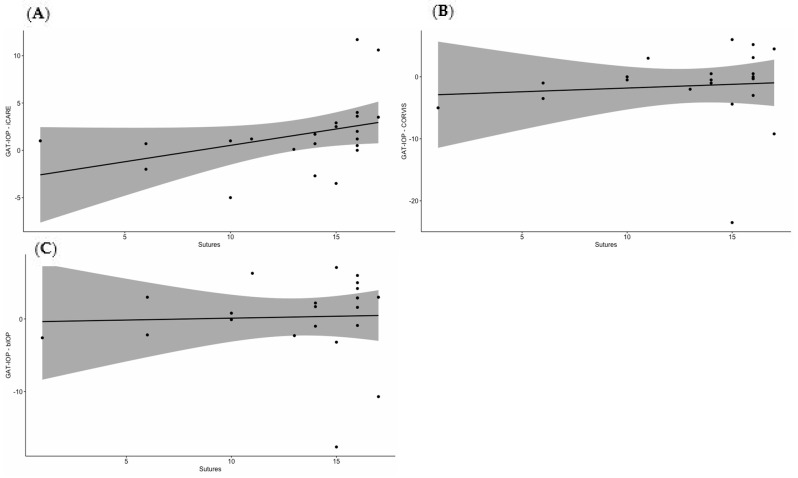
Kendall’s Tau rank correlation analysis plots demonstrating the relationship between the number of sutures and the differences in intraocular pressure (IOP) measurements between Goldmann applanation tonometry (GAT) and (**A**) iCare, (**B**) uncorrected Corvis ST IOP (CVS-IOP), and (**C**) biomechanically compensated IOP obtained by Corvis ST (bIOP). The solid line represents the overall trend of association between the number of sutures and ΔIOP. The moderately upward slope in Graph A indicates that as the number of sutures increased, the ΔΙOP between iCare and GAT also tended to increase.

Finally, Kendall’s Tau rank correlation analysis examining the relationship between the number of sutures and the differences in IOP measurements by GAT compared to the other tonometers (Figure 2) revealed a statistically significant moderate positive correlation only in the ΔIOP between GAT and iCare (*p* = 0.0079). As the number of remaining sutures increased, the ΔIOP also tended to increase (Tau = 0.430). Cases with a continuous suture were excluded from this analysis.

## 4. Discussion

Although the differences in IOP measurements between GAT and other tonometers in post-penetrating keratoplasty patients were generally not statistically significant in this study, the Bland–Altman analysis revealed a relatively wide LoA for both iCare and CVS-IOP, indicating substantial variability in individual measurements. The bIOP showed the closest agreement with GAT-IOP, exhibiting the narrowest LoA.

Only a few studies in the literature have assessed the agreement of IOP measurements between different devices in eyes following penetrating keratoplasty. Salvetat et al. reported poor agreement between the iCare and GAT readings, with iCare significantly underestimating IOP in post-PK eyes without corneal oedema. However, they concluded that when both devices show high IOP readings in grafts, it may indicate truly elevated IOP in the eye [35]. Unlike the findings of the previous study, our results show good agreement between iCare and GAT-IOP, with iCare measurements exhibiting a slight positive bias compared to GAT-IOP.

Corvis ST and its bIOP estimate have been regarded as a significant milestone in the follow-up of patients who have undergone various refractive corneal surgeries [32,36,37]. However, its potential application in post-PK patients has not yet been investigated. Eliasy et al. previously reported that the bIOP estimates closely approximate the manometric IOP measurements conducted on cadaveric eyes, whereas CVS-IOP does not [28]. In more recent in vivo studies involving both healthy individuals and primary open-angle glaucoma patients Corvis ST demonstrated good repeatability in IOP readings and reduced interobserver variability. However, its measurements were not directly comparable to GAT readings [38,39,40]. Vinciguerra et al. found that the bIOP measurements were significantly lower than both GAT-IOP and GAT-IOP adjusted for CCT in normal and ocular hypertension eyes, while being significantly higher in patients with high-tension and normal-tension glaucoma [41]. Ramm et al. observed good agreement between bIOP and CCT-corrected GAT-IOP readings in healthy eyes [42]. Our study demonstrated good agreement between both CVS-IOP and bIOP with GAT-IOP readings in post-PK eyes, with CVS-IOP tending to underestimate and bIOP slightly overestimating IOP compared to GAT-IOP.

There is an observed trend of higher CCT in post-PK eyes; nevertheless, this has not been proven to be statistically significant [14]. The influence of CCT on GAT IOP measurements in normal eyes is well recognized. Thicker corneas tend to offer greater resistance to deformation during tonometry, potentially leading to an overestimation of IOP readings. However, this relationship is not linear as previously suggested, and CCT-based IOP adjustment nomograms are often flawed [19,30]. Rebound tonometry has also been shown to be influenced more by CCT compared to GAT [22,24]. In post-keratoplasty eyes, however, the relationship between CCT and IOP measurements may be less significant or even non-existent [18,35,43]. Although our study revealed a moderate negative correlation between CCT and bIOP, we did not identify a linear relationship between CCT and the difference in IOP readings from different devices.

Sutures at the graft–host interface after PK can potentially affect corneal properties such as astigmatism and corneal curvature; however, their influence on IOP measurement has been shown minimal [14]. Kandarakis et al. found GAT to be less effective in measuring IOP in grafts with more than four remaining sutures, as well as in recent PKs (less than one year) and regrafts [13]. In our study, a statistically significant correlation was observed only in the ΔIOP between GAT and iCare, indicating that as the number of sutures increased, the ΔIOP also tended to increase.

Corneal biomechanical properties, such as viscosity, elasticity, and viscoelasticity, can be altered in glaucoma [27]. Anti-glaucomatous eye drops have also been shown to influence the corneal extracellular matrix, potentially affecting corneal rigidity and, subsequently, IOP readings in normal eyes [44]. In our sample, no significant differences in tonometer readings were found with the use of antiglaucoma medication.

Our study has several limitations. First, it remains uncertain which IOP measurement most accurately reflects the true value. While we compared IOP measurements obtained from iCare and Corvis ST with GAT, the most commercially available tonometer and widely referred to as the gold standard, it is important to note that GAT-IOP may not represent a true IOP value. The small sample size also poses a limitation; therefore, further research involving a larger cohort is needed to obtain statistically reliable results. Finally, corneal biomechanical properties, such as viscosity, elasticity, and viscoelasticity, can be altered in various ocular disorders, including keratoconus, ocular hypertension, glaucoma, and diabetes mellitus [27,41,45]. These patients were not excluded from our sample, which could be a confounding factor in the results. Nonetheless, the findings in this study represent ‘real world’ outcomes and can be translated to routine clinical practice.

## 5. Conclusions

In conclusion, good agreement was found between iCare, CVS-IOP, bIOP, and GAT-IOP readings with the comparison between GAT-IOP and bIOP yielding the narrowest 95% LoA. These findings are promising and suggest that all three tonometers are applicable for clinical IOP measurement in eyes that have undergone PK, despite each technique operating on a distinct principle and mechanism. The difference between GAT-IOP and iCare readings appeared to be influenced by the number of sutures at the graft–host interface. Higher CCT values were associated with lower bIOP readings; however, the differences in tonometer readings compared to GAT-IOP were not found to be influenced by the CCT.

There is still no consensus in the literature regarding which of the methods mentioned above constitutes a consistent and reliable alternative. Further research is yet to be conducted, particularly involving Corvis ST and its bIOP estimate, which is less dependent on factors such as age, CCT, and corneal biomechanical response, and has been shown to closely approximate true IOP in previous studies with ex vivo eyes. Finally, it is important to note that, as there is no consistent data on the relationship between IOP measurements obtained from different devices, they should not be used interchangeably in clinical practice.

## Figures and Tables

**Table 1:** Demographic data of patients.

Age	66.1 ± 12.2 years *
Sex	15 females|10 males
Laterality	RE 12|LE 13
CDVA	0.74 ± 0.5 logMAR
Time since PK	14.4 ± 13.5 months *

* mean ± standard deviation, CDVA: Corrected Distance Visual Acuity, PK: penetrating keratoplasty, RE: right eye, LE: left eye.

**Table 2:** Average and standard deviation (SD) of intraocular pressure (IOP) measurements across various tonometers.

	Mean ± SD (mmHg)	Range (mmHg)
GAT-IOP	15.9 ± 6.3	8.5–37.5
iCare	14.6 ± 4.2	10.0–25.8
CVS-IOP	17.4 ± 7.4	9.5–37.0
bIOP	15.7 ± 7.2	6.2–33.7

GAT-IOP: mean Goldmann IOP, CVS-IOP: uncorrected Corvis ST IOP, bIOP: biomechanically compensated IOP obtained by Corvis ST.

**Table 3:** Correlation coefficients amongst different tonometers and CCT (central corneal thickness).

	Tau Value	z	*p*
GAT-IOP	−0.1623782	−11062	0.2686
iCare	−0.02711864	−0.18735	0.8514
CVS-IOP	0.05093386	0.35137	0.7253
bIOP	−0.3905812	−2.7136	0.006655

GAT-IOP: mean Goldmann IOP, CVS-IOP: uncorrected Corvis ST IOP, bIOP: biomechanically compensated IOP obtained by Corvis ST.

**Table 4:** Correlation coefficients amongst the difference of IOP measurements (ΔIOP) between different tonometers and CCT.

GAT-IOP Minus	Tau Value	z	*p*
iCare	−0.2027039	−14.043	0.1602
CVS-IOP	−0.153324	−1.0549	0.2915
bIOP	0.1720089	11.934	0.2327

GAT-IOP: mean Goldmann IOP, CVS-IOP: uncorrected Corvis ST IOP, bIOP: biomechanically compensated IOP obtained by Corvis ST.

## Data Availability

The raw data supporting this study’s findings are available from the corresponding author upon request.
